# Electronic, Structural, and Optical Properties of Mono-Doped and Co-Doped (210) TiO_2_ Brookite Surfaces for Application in Dye-Sensitized Solar Cells—A First Principles Study

**DOI:** 10.3390/ma14143918

**Published:** 2021-07-14

**Authors:** Ratshilumela S. Dima, Lutendo Phuthu, Nnditshedzeni E. Maluta, Joseph K. Kirui, Rapela R. Maphanga

**Affiliations:** 1 Next Generation Enterprises and Institution Cluster, Council for Scientific and Industrial Research, P.O. Box 395, Pretoria 0001, South Africa; SDima@csir.co.za (R.S.D.); rmaphanga@csir.co.za (R.R.M.); 2 Department of Physics, University of Venda, P/Bag X 5050, Thohoyandou 0950, South Africa; lutendo.phuthu@univen.ac.za (L.P.); Joseph.Kirui@univen.ac.za (J.K.K.); 3 National Institute for Theoretical Physics (NITheP), Gauteng 2000, South Africa

**Keywords:** on-metal, brookite TiO_2_, doping mechanism, optical properties, density functional theory

## Abstract

Titanium dioxide (TiO_2_) polymorphs have recently gained a lot of attention in dye-sensitized solar cells (DSSCs). The brookite polymorph, among other TiO_2_ polymorphs, is now becoming the focus of research in DSSC applications, despite the difficulties in obtaining it as a pure phase experimentally. The current theoretical study used different nonmetals (C, S and N) and (C-S, C-N and S-N) as dopants and co-dopants, respectively, to investigate the effects of mono-doping and co-doping on the electronic, structural, and optical structure properties of (210) TiO_2_ brookite surfaces, which is the most exposed surface of brookite. The results show that due to the narrowing of the band gap and the presence of impurity levels in the band gap, all mono-doped and co-doped TiO_2_ brookite (210) surfaces exhibit some redshift. In particular, the C-doped, and C-N co-doped TiO_2_ brookite (210) surfaces exhibit better absorption in the visible region of the electromagnetic spectrum in comparison to the pure, S-doped, N-doped, C-S co-doped and N-S co-doped TiO_2_ brookite (210) surfaces.

## 1. Introduction

The answer to the growing energy demand, sustainability of energy sources and conservation of the environment for the world might lie in the ability to capture and utilize renewable energy sources. Even when the cost of solar energy is decreasing, the dye-sensitized solar cells (DSSCs) have attracted more attention as one of the most popular solar cells as they have been shown to have a simple fabrication process, low manufacturing cost and relatively high conversion efficiencies [1,2].

In the DSCCs, the TiO_2_ semiconductor is very important for the efficiency of the cell as the dye molecules adsorb on its surface. The dye molecules are used for the photonadsorption and they are the source of the photocurrent of the solar cells [3,4,5,6]. However, the electron band gap of TiO_2_ is relatively large (3.2 eV for anatase, 3.0 eV for rutile and 3.4 eV for brookite) [7,8,9,10]. To improve the efficiency of the DSSC, the requirements will include: (1) reduction of the TiO_2_ band gaps, so that its absorption properties will match the visible solar spectrum, and (2) modification of the sensitizer or dye so that it can absorb the over most of the entire solar spectrum [2]. Therefore, the modification of different electronic and optical properties of TiO_2_ polymorphs (anatase, rutile and brookite) is very important for the application of DSCC and related technologies such as photo-catalysis. Several demonstrations of the performance of DSSCs based on the photoanode made up of sulfur-doped, vanadium-doped, cobalt-doped TiO_2_ nanoparticles synthesized using different methods have been reported [11,12,13].

Improving the visible light response of oxide semiconductor materials by reducing the band gap energy has become a universal research focus in the photocatalytic field using both experimental and theoretical techniques. Although TiO_2_ is an attractive semiconductor for DSCCs, it can only make use of less than 5% of the solar spectrum in its pristine state owing to its large band gap [1,6,7,8,10].

Different experimental and theoretical work has been performed to improve the photocatalysis of different surfaces of anatase and rutile TiO_2_ through mono-doping and co-doping with different metals and non-metals, such as; vanadium, tantalum, nitrogen, carbon, platinum, sulfur, etc [11,12,14,15,16,17,18]. It was reported that N-doped TiO_2_ shows a relatively high level of visible light activity. Co-doping of nitrogen and other elements into the TiO_2_ lattice has also been reported [19,20,21,22].

Chen et al. [23] used density functional calculations to analyze the geometrical parameters, the density of states, charge densities, relative dielectric functions, and UV–Vis absorption spectra of N-S co-doped rutile TiO_2_ with two doping mechanisms (substitution and interstitial) to obtain a more efficient (N-S) co-doping scheme. They discovered that, when compared to (N-S) substitution, co-doped TiO_2_, (N-S) interstitial co-doped TiO_2_ exhibits a more obvious red-shift of the absorption edge due to a smaller band gap. They also reported shallow impurity levels coupling with the top of the valence band. Their calculated UV–Vis’s absorption spectra revealed that TiO_2_ with (N-S) interstitial co-doping has significantly higher photocatalytic activity in the visible light region.

Guo et. al. [24] reported that the C–N–S co-doped TiO_2_ mixed crystal exhibited a unique isoband gap characteristic and a prominent red-shift in the ultraviolet and visible absorption spectra and a much narrower band [24]. The effects of doping different materials can be evaluated easily by the calculation of electronic structure and related properties.

Brookite, one of the three crystallographic types of TiO_2_, has received much less attention and understanding than rutile and anatase, owing to the difficulty in producing large quantities of high-quality material. However, efficient methods for synthesizing high purity brookite TiO_2_ have recently been developed [25]. This has sparked increased interest in the physicochemical properties and potential applications of this TiO_2_ polymorph, which were found to exhibit higher activity in photocatalysis and catalytic reactions involving TiO_2_-assisted metal clusters in a few cases than rutile and anatase. Li et al. [26] reported that the commonly exposed brookite (210) surface is more reactive than the ubiquitous anatase (101) surface and might be useful in catalytic and photocatalytic applications; this was performed using the density functional theory (DFT) calculations. In this paper, we report a comparative density functional theory study of nonmetals (C, S and N) mono-doping and co-doping of the brookite (210) surface. The main discussion is focused on how the structural, electronic, and optical properties of the (210) brookite surface change owing to non-metals (C, S and N) mono-doping and co-doping. This paper will report for the first time on the DFT study of the (C, S, and N) mono- and co-doped (210) brookite surfaces. As a result, a novel approach will be presented in this work that will aid in the design of structures with increased photocatalytic activity when exposed to visible light from the surface of brookite (210).

## 2. Materials and Methods

To determine the structural, electronic, and optical properties of mono-doped, co-doped, and un-doped (210) brookite surfaces, density functional theory (DFT) calculations were performed using the plane-wave pseudopotential method. The exchange-correlation functional was described using the generalized gradient approximation (GGA) in the Perdew–Burke–Ernzerhof (PBE) scheme. All calculations were performed in BIOVIA Inc’s Materials Studio, San Diego, CA, USA, using the CASTEP code [27]. According to the convergence test, the cut-off kinetic energy of plane waves is 650 eV. A Monkhorst–Pack mesh was used for the Brillouin zone k-point sampling, 4 × 4 × 2. The convergence criteria for structural optimization were set to medium quality, with tolerances of 2.0106 eV/atom, 2.0105 eV/atom, 0.05 eV/atom, and 2.0103 eV/atom for the self-consistent field (SCF), energy, maximum force, and maximum displacement, respectively. The calculated equilibrium geometry was used to derive the (210) surface structure of brookite from an unreconstructed bulk structure slab model. The (210) surface slab model had a vacuum thickness of ten. The relaxation of these surfaces was accomplished by optimizing all the slab’s layers. The surfaces of the (210) brookite were mono-doped by replacing one O atom with either (C, N, or S) and co-doped by replacing two O atoms with either (C-S, C-N, or S-N) (substitutional doping). The orbital electrons involved in the calculations have the following valence electrons: Ti: 3s23p63d24s2, O: 2s^2^2p^4^, N: 2s^2^2p^3^, S: 3s^2^3p4, C: 2s^2^2p^2^.

## 3. Results and Discussion

### 3.1. Bulk TiO_2_ Brookite

#### 3.1.1. Structural Properties

**Figure 1:**
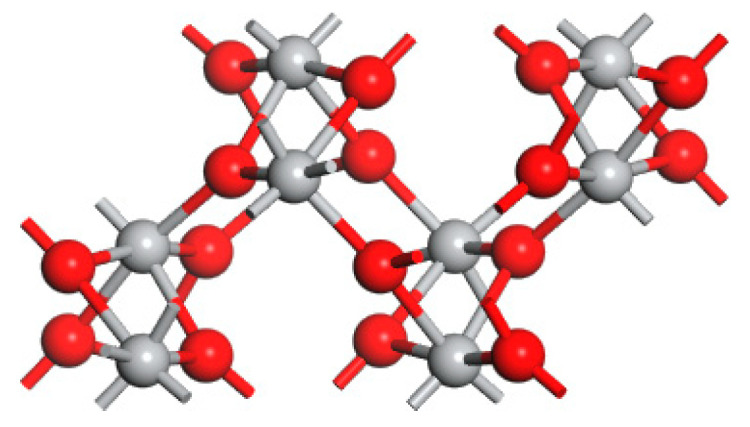
The bulk structure of TiO_2_ brookite. The grey spheres represent Ti atoms while the red spheres represent O atoms.

The crystal structure of brookite TiO_2_ (orthorhombic, space group Pbca) was visualized as shown in Figure 1. Brookite’s unit cell contains 24 atoms, eight of which are titanium (Ti) and sixteen are oxygen (O) atoms with eight formula units. The brookite is a TiO_6_ octahedron, where Ti binds with six O-atoms. The Ti-O bond length ranges from 1.8534 to 2.063 Å.

The experimental and calculated structural data for bulk brookite are presented in Table 1. The calculated results are in agreement with both the experimental and reported theoretical results [9,10]. The obtained lattice parameters deviate from experimental data by 0.1967% along the a-axis, 0.203% along the b-axis and 0.196% along the c-axis. In comparison with the previous theoretical results reported, the calculated results are more accurate relative to experimental data, and thus validating the starting models and method used. The results show that the atomic internal coordinates also vary isotopically at different positions. The theoretical determination of the stability and structure of materials under conditions that are difficult to reproduce in the laboratory is an added value of the present calculations.

#### 3.1.2. Electronic Properties

Using the measured lattice parameters listed in Table 1, the band structure of brookite TiO_2_ was built along with the appropriate high-symmetry directions of the corresponding irreducible Brillouin zone. A 2.353 eV measured energy band gap for bulk brookite TiO_2_ was obtained. The band gap value obtained in this study is similar to that reported by Mo and Ching [8], who calculated a value of 2.20 eV using the self-consistent orthogonalized linear combination of atomic orbitals method to arrive at their result. Due to the limitations of DFT, both values were found to be underestimated as compared to the experimentally tested value of 3.40 eV. The restriction is due to a discontinuity in the exchange-correlation potential that is not taken into account in the DFT calculations.

**Figure 2:**
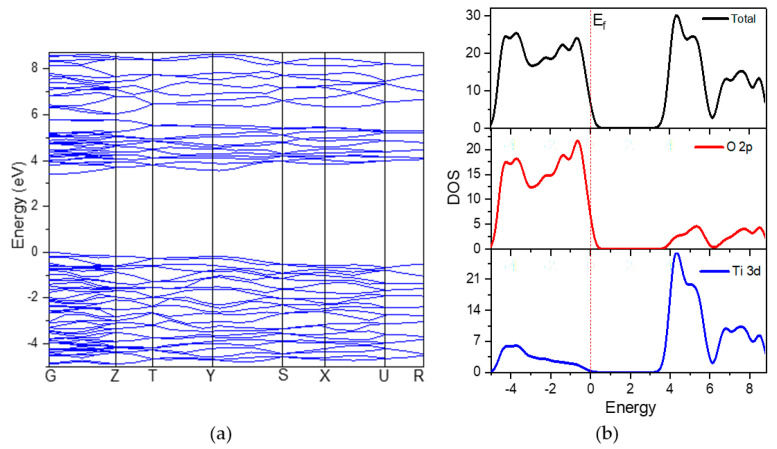
Calculated (**a**) band structure and (**b**) density of states of bulk brookite TiO_2_.

The debate over the energy difference, on the other hand, would be unaffected because only relative energy shifts are of interest. To compensate for the underestimation of the band gap, the scissor operation of 1.047 eV was used in this study [28]. The scissors scheme aligns the theoretical valence band energy (VBE) and conduction band energy (CBE) with their experimental equivalents by stretching the theoretical gap states over the experimental gap using a “scissors” process. The experimental value for the scissor operation is 3.40 eV, as stated by Koelsch et al. [7] in their study of brookite nanoparticles dispersed in water. The band gap value after the scissor operation is 3.42 eV, as shown in Figure 2. Both the conduction band minimum (CBM) and the valence band maximum (VBM) are located at G. Therefore, brookite TiO_2_ is considered to be a direct band gap semiconductor. The upper valence band (VB) is lying in the range of 0 to 87eV consisting of O *2p* orbitals hybridized with Ti *3d* orbitals. The bottom of the conduction band (CB) at −1.24 eV is composed of Ti *3d* orbitals.

### 3.2. (210). TiO_2_ Surface

#### 3.2.1. Structural Properties

The (210) surface is a step surface composed of oxygen ions that are two-fold coordinated in both the top and bottom layers. Additionally, it contains a mixture of two- and three-fold coordinated oxygen ions in the middle layers. Titanium ions are coordinated five-fold and six-fold. While terminating with both oxygen and titanium, the atoms in these structures have some cleaved bonds in the termination positions. The surface structures of both undoped and doped (210) brookite were optimized by relaxing surface atoms to eliminate surface atomic tension.

#### 3.2.2. Electronic Properties

The energy band gap of pure TiO_2_ (210) surface is 3.54 eV and it is 1.161 eV larger than that of the bulk system. The larger band gap of brookite (210) indicates that the electron’s distribution probability is greatest near the surface, implying that the electron is constrained near the surface. This type of electronic state is referred to as a surface state, and the associated energy level is referred to as the surface level.

**Figure 3:**
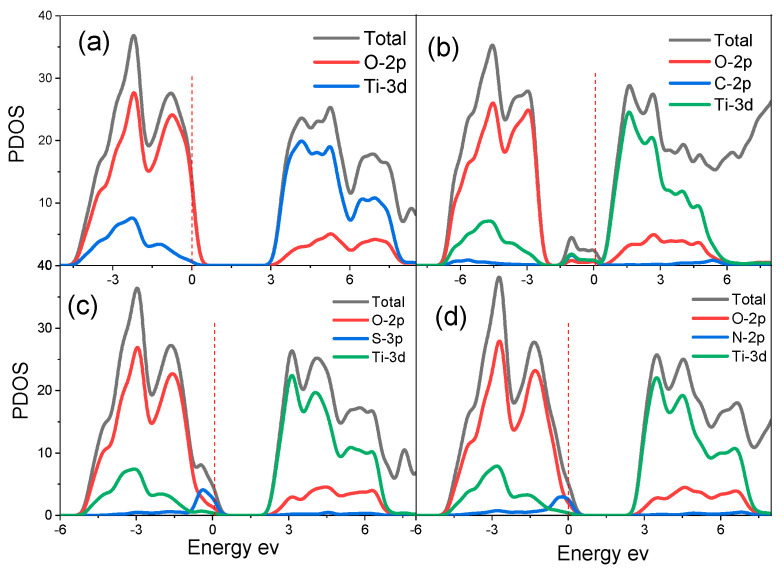
Calculated density of states (DOS) of (**a**) pure, (**b**) C-doped, (**c**) S-doped, and (**d**) N-doped bulk brookite TiO_2_.

Figure 3a illustrates unequivocally that no surface level appears near the Fermi level on the (210) surface, indicating that no surface states exist. These modifications are primarily due to the redundant electronics of the brookite TiO_2_ (210) surface transfer to the other atoms. The conduction band energy ranges from 2.870 to 6.14 eV and a width of 3.27 eV, with the Ti *3d* track accounting for the majority of the energy and a small amount of O *2p* track accounting for the remainder. The valence band energy near the Fermi level is between 0.509 and 5.005 eV, which is also attributed to the hybridization of the O *2p* and Ti *3d* orbits. In comparison to the bulk system’s band, the brookite TiO_2_ (210) surface band is smoother, and the corresponding surface state density peak is larger. Similar results were reported by Beltran et al. [10] on (110) brookite surface. This result is due to two factors: first, the system contains a variable number of atoms; and second, the band gap width changes, thereby strengthening the surface electronic density of states and localization.

In the case of N-, C- and S-doped brookite TiO_2_ (210) surfaces, the band gaps were determined as 3.042 eV, 1.363 eV and 2.656 eV and represented in Figure 3b–d, respectively. A smaller band gap value of the doped systems in comparison with the undoped (210) suggests that doping with N, C and S results in the narrowing of the band. The curves also show that N-doped (210) surface caused a small narrowing of the band gap. The lowest band gap was observed in C-doping, giving the highest narrowing of the band gap. This narrowing of the doped (210) surfaces arises from the formation of the impurity energy levels (IELs), which are a result of hybridizing with O *2p* states or Ti *3d* states and C *2p* states. The IELs contribute to the separation of photo-generated electron–hole pairs and favor the migration of photoexcited carriers and the process of photocatalysis. The impurity energy levels are located below the CB and overlap with the VB. These kinds of IELs could act as trap centers for photoexcited holes, and hence reduce the recombination rate of charge carriers.

**Figure 4:**
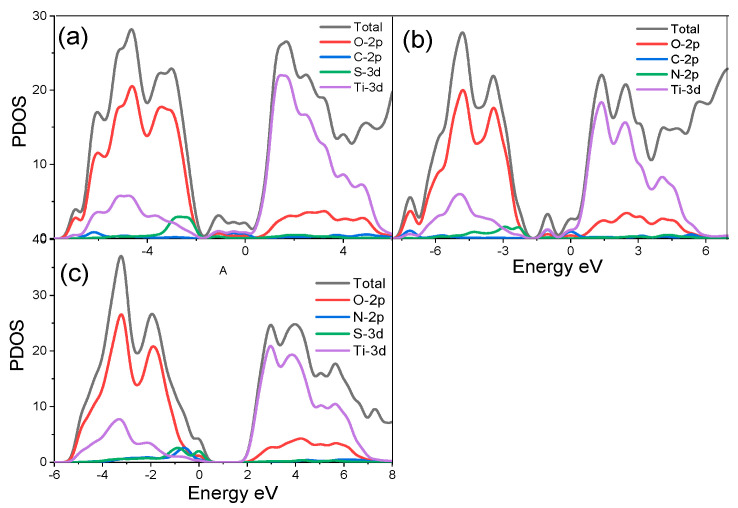
Calculated density of states (DOS) of (**a**) C,S-doped, (**b**) C,N-doped and (**c**) S,N-doped (210) surface.

Three different co-doped surface structures were modelled, and their DOSs are shown in Figure 4.

Similarly, the C-N, N-S and S-C co-doping of (210) surface narrowed the band gaps of the surfaces and yielded energy band gap values of 0.920 eV, 2.37 eV and 1.456 eV, respectively. The N-C and S-C co-doping of (210) surface shifted the semiconductor from p-type to n-type. Again, the narrowing of the band gap is due to the formation of the IELs, which are a result of hybridizing with O *2p* states or Ti *3d* states and C *2p* states. Further exploration of the electronic properties of (210) brookite surface from Ti, O, C, N and S atoms can be obtained from the Mulliken population analysis. The partial atomic charges of Ti, O, C, N and S atoms are shown in Table 2.

The results from C-N, N-S and S-N co-doping of (210) surface show that the partial charges of about 1.15 to 1.35e for Ti, −0.45 to −0.79e for O, −0.30 to −0.52e for C, −0.71 to −0.80e for N and −0.29 to −0.42e for S, these charges are larger than +1 for Ti and less than −0.6 for O and S. The present net charges (e) of Ti, O, C, N and S atoms indicate a charge transfer between the atoms.

### 3.3. Optical Properties

The dielectric function and optical absorption coefficient of various doped systems are calculated using a non-polarization model, which provides additional insight into the doping effect. The dielectric function serves as a bridge between interband transition microphysical processes and solid-state electronic structure, allowing solids to reveal their optical properties. The dielectric function in the virtual $i\varepsilon_{2}\left(\omega \right)$ shows the electrons in the electromagnetic radiation perturbation effect from low level to high-level processes, namely the absorption of interband transitions. The imaginary part of the dielectric function indicates the material’s absorptive capacity for photons, the larger the imaginary part, the greater the possibility of photon absorption. The electronic instability at a high energy level will spontaneously shift to a low energy level, which is represented by the real part of the dielectric function $\varepsilon_{1}\left(\omega \right)$, depicts the material’s ability to reflect light. As a result, the dielectric function directly reveals the material’s absorption and reflection of light. Within the range of linear optical response, the macroscopic optical response function of the solid is typically described by the complex dielectric function of light. The frequency-dependent dielectric function is given by Equation (1):

(1) $$\varepsilon \left(\omega \right)=\varepsilon_{1}\left(\omega \right)+i\varepsilon_{2}\left(\omega \right).$$

The dielectric function is closely related to the electronic band structure. It fully describes the optical properties of any homogeneous medium at all photon energies. The imaginary part $\varepsilon_{2}\left(\omega \right)$ of the complex dielectric function is obtained from the momentum matrix elements between the occupied and unoccupied electronic states. It is calculated using the analytical expression in Equation (2):

(2) $$\varepsilon_{2}\left(\omega \right)=\frac{2e^{2}\pi }{\Omega \varepsilon_{0}}\underset{k,v,c}{\sum }|\langle \psi_{k}^{c}|\hat{u}*\vec{r}|\langle \psi_{k}^{v}{\|}^{2}\delta \left(E_{k}^{c}-E_{k}^{v}-E\right)$$

where *ω* is the frequency of light, *e* is the electronic charge, *û* is the vector defining the polarization of the incident electric field, $\psi_{k}^{c}$ and $\psi_{k}^{v}$ are the conduction and valence band wave functions at *k*, respectively.

**Figure 5:**
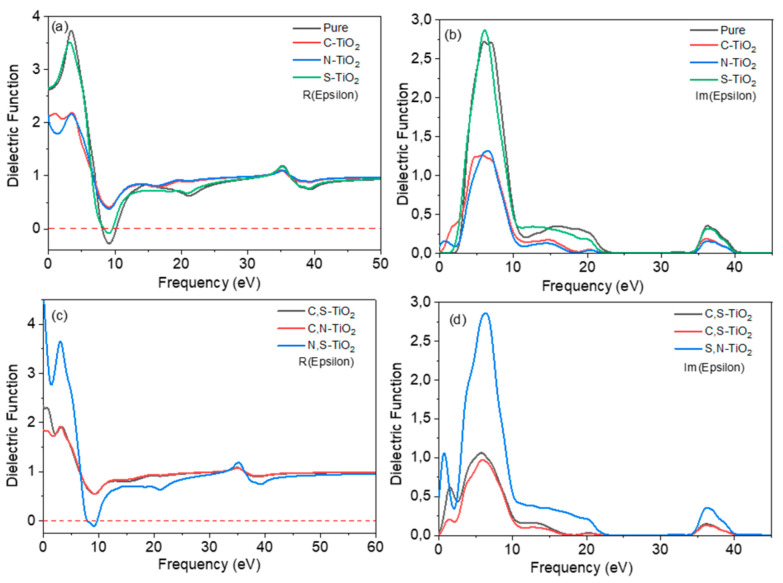
Real parts of the dielectric function of (**a**) pure, (C, N and S) mono-doped and (**b**) (C-N, N-S and S-N) co-doped and imaginary parts of the dielectric function of (**c**) pure, (C, N and S) mono-doped, and (**d**) (C-N, N-S and S-N) co-doped.

The dielectric function describes the polarization and absorption of the material. The real and imaginary parts of the dielectric function versus photon energy change curve are shown in Figure 5a–d. The dielectric constant $\varepsilon_{0}$ of pure as well as, C-, N- and S-doped surfaces from the standard functional presented the values of 2.629, 2.143, 2.142 and 2.660, respectively. For N-S, N-C and S-C co-doped brookite systems, the dielectric constant ε_0_ from the standard functional were 2.330, 1.851 and 5.085, respectively. The real part $\varepsilon_{1}\left(\omega \right)$ of the dielectric function shows the highest peak intensity at 2.717, 1.252, 1.314 and 2.865 for pure, C-doped, N-doped and S-doped, respectively. For N-S, N-C and S-C co-doped surfaces, the real parts $\varepsilon_{1}\left(\omega \right)$ of the dielectric function show the highest peak intensity at 1.062, 0.973 and 2.858, respectively.

For the imaginary part of all the surfaces, the peaks associated with $\varepsilon_{0}$ less than 4 eV are due to intraband transitions and this peak for mono doping was found to have lower intensity as compared to those of co-doped surfaces. These peaks belong to the electronic transition from Ti 3*d* to O 2*p* states and the new 2*p* from C and N and 3*p* from S at the conduction band and valence band. These peaks of the imaginary part of the dielectric function are related to the electron excitation. It can be observed that the value of the imaginary part of the dielectric function is zero between ~24 and 35 eV for all the mono-doped and co-doped (210) brookite surfaces. Therefore, these materials become transparent in this region and above 50 eV. The real part of the dielectric function falls below zero at about ~9.8 eV for pure, S-doped and N-S co-doped (210) brookite surfaces.

**Figure 6:**
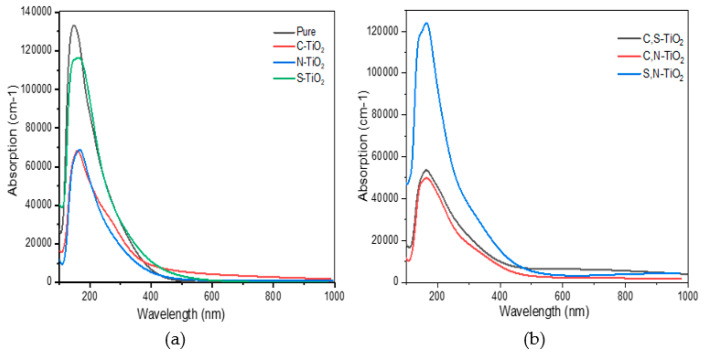
Optical absorption curves of the (**a**) (C, N and S) mono doped and (**b**) (C-S, C-N and N-S) (210) doped brookite surface.

The absorption coefficient gives important information about solar energy conversion efficiency and how far light of a specific frequency can penetrate into the material before being absorbed. Figure 6 reveals that the absorption edges of mono-doped and co-doped (210) surfaces, move absorption capability toward the longer wavelength, implying that there is an enhancement of visible light absorption. C-doped and N-S doped has stronger light absorption in both the visible region and the near-infrared region as compared to pure, (C-S) mono-doped, C-N- doped and N-S- doped (210) brookite surfaces. Thus, C-doped and C-S-doped surfaces have higher photocatalytic activity. The doping creates IELs within the band structures, which can act as a “step” that reduces the electronic transition energy. Thus, more electrons can be excited and hence there is an enhanced probability that photoexcited electrons can migrate to the surface. Meanwhile, IELs could also act as a separating center, where the photoexcited electron–holes can separate rapidly and effectively, thus promoting the carrier’s diffusion and enhance the charge carrier lifetime.

## 4. Conclusions

In this study, we systematically analyzed geometrical parameters and the electronic structures of bulk TiO_2_ brookite, using density functional calculations and the results are consistent with the experimental and other theoretical work. The analysis of electronic structures and optical properties for pure, C-doped, S-doped, N-doped, C-S co-doped, C-N-doped and N-S co-doped TiO_2_ brookite (210) surfaces were conducted. The results showed that due to the narrowing of the band gap and the presence of impurity levels in the band gap, all mono-doped and co-doped TiO_2_ brookite (210) surfaces exhibit some redshift. In particular, the C-doped, S-N co-doped and C-N co-doped TiO_2_ brookite (210) surfaces exhibit better absorption in the visible region of the electromagnetic spectrum.

For the C-doping and S-N co-doping, impurity states appear in the valence band, coupling with the top of the valence band edge, which not only narrows the band gap but also effectively captures photoinduced electrons and reduces charge carrier recombination in the crystal lattice of the brookite (210) surfaces. The reduction in the recombination may arise because C-doping and S-N co-doping generate surface oxygen vacancies that serve as traps for photogenerated electrons and reduced electron–hole pair recombination. As a result, C-doping and S-N co-doping suggest enhanced photocatalytic activity.

## Figures and Tables

**Table 1:** Calculated lattice parameters for bulk brookite TiO_2_ compared with experimental and previous theoretical results.

Lattice Parameter	Literature Experimental [9]	Literature Calculations [10]	This Work Calculations
a (Å)	9.184	9.157	9.166
b (Å)	5.447	5.430	5.436
c (Å)	5.145	5.122	5.135
*V* (Å)^3^	25,8.40	27,0.37	25,5.86

**Table 2:** Mulliken population analysis of (210) brookite surface for s, p, and d orbitals.

		s	p	d	Total	Charge
Pure	O	1.89	4.90	0	6.79	−0.76
	Ti	2.21	6.24	2.26	10.69	1.32
C-doped	O	1.89	4.90	0	6.79	−0.45
	C	1.4	3.06	0	4.52	−0.52
	Ti	2.27	6.27	2.31	10.85	1.32
N-doped	O	1.89	4.90	0	6.79	−0.56
	N	1.78	3.93	0	5.71	−0.71
	Ti	2.23	6.25	2.29	10.77	−1.33
S-doped	O	1.89	4.90	0	6.79	−0.54
	S	1.87	4.54	0	6.42	−0.42
	Ti	2.26	6.32	2.32	10.90	1.35
C- S-doped	O	1.89	4.91	0	6.80	−0.54
	C	1.50	3.01	0	4.51	−0.31
	S	1.88	4.44	0	6.35	−0.32
	Ti	2.29	6.30	2.49	11.08	1.34
C-N-doped	O	1.89	4.90	0	6.79	−0.77
	C	1.51	2.78	0	4.30	−0.30
	N	1.74	4.06	0	5.08	−0.80
	Ti	2.25	6.27	2.38	10.90	−1.19
N-S-doped	O	1.90	4.90	0	6.80	−0.79
	N	1.76	4.03	0	5.79	−0.79
	S	1.91	4.38	0	6.29	−0.29
	Ti	2.25	6.31	2.36	10.92	1.15

## Data Availability

Not applicable.

## References

[1] Magne C., Dufour F., Labat F., Lancel G., Durupthy O., Cassaignon S., Pauporté T. (2012). Effects of TiO_2_ nanoparticle polymorphism on dye-sensitized solar cell photovoltaic properties. J. Photochem. Photobiol. A.

[2] Serrano L.A., Park K., Ahn S., Wiles A.A., Hong J., Cooke G. (2019). Coplanar Donor-π-acceptor dyes featuring a furylethynyl spacer for dye-sensitized solar cells. Materials.

[3] Landmann M., Rauls E., Shmidt E.G. (2012). The electronic structure and optical response of rutile, anatase and brookite TiO_2_. J. Condens. Matter. Phys..

[4] Park J.Y., Lee C., Jung K.W., Jung D. (2009). Structure related photocatalytic properties of TiO_2_. Bull. Korean Chem. Soc..

[5] Samat M.H., Yahya M.Z.A., Taib M.F.M., Samsi N.S., Hussin N.H., Ali A.M.M., Yaakob M.K., Aziz S.S.S.A. (2016). First-principles study on structural, electronic, and optical properties of TiO_2_ for dye-sensitized solar cells photoanode. Mater. Sci. Forum.

[6] Dima R.S., Maluta N.E., Maphanga R.R., Sankaran V. (2017). Computational study of TiO_2_ brookite (100), (010) and (210) surface doped with ruthenium for application in dye sensitised solar cells. J. Phys. Conf. Ser..

[7] Koelsch M., Cassaignon S., Guillemoles J.F., Jolivet J.P. (2002). Comparison of optical and electrochemical properties of anatase and brookite TiO_2_ synthesized by the sol–gel method. Thin Solid Films.

[8] Mo S., Ching W. (1995). Electronic and optical properties of three phases of titanium dioxide: Rutile, anatase, and brookite. Phys. Rev. B Condens. Matter..

[9] Meagher E.P., Lager G.A. (1979). Polyhedral thermal expansion in the TiO_2_, polymorphs: Refinement of the crystal structures of rutile and brookite at high temperature. Canad. Mineral..

[10] Beltrán A., Gracia L., Andrés J. (2006). Density functional theory study of the brookite surfaces and phase transitions between natural titania polymorphs. J. Phys. Chem. B.

[11] Gao P., Yang L., Songtao X., Wang L., Guo W., Lu J. (2019). Effect of Ru, Rh, Mo, and Pd adsorption on the electronic and optical properties of anatase TiO_2_(101): A DFT investigation. Materials.

[12] Dima R.S., Maluta N.E., Maphanga R.R. (2020). First-principles investigation of Ru-and Pt-doped TiO_2_ brookite surfaces. Int. J. Electrochem. Sci..

[13] Wang T., Shen D., Xu T., Jiang R. (2017). Photocatalytic degradation properties of V-doped TiO_2_ to automobile exhaust. Sci. Total Environ..

[14] Li S., Huang J., Ning X., Chen Y., Shi Q. (2018). First-principles study of Mn-S co doped anatase TiO_2_. MRX.

[15] Samat M.H., Ali A.M.M., Taib M.F.M., Hassan O.H., Yahya M.Z.A. (2016). Hubbard U calculations on optical properties of 3d transition metal oxide TiO_2_. Results Phys..

[16] Yu Q., Jin L., Zhou C. (2011). Ab initio study of electronic structures and absorption properties of pure and Fe^3+^ doped anatase TiO_2_. Sol. Energy Mater. Sol. Cells..

[17] Zhang R., Zhao J., Yang Y., Lu Z., Shi W. (2016). Understanding electronic and optical properties of La and Mn co-doped anatase TiO_2_. Comput. Condens. Matter..

[18] Dubey R.S., Jadkar S.R., Bhorde A.B. (2021). Synthesis and characterization of various doped TiO_2_ nanocrystals for dye-sensitized solar Cells. J. Am. Chem. Soc..

[19] Huang L.H., Sun C., Liu Y.L. (2007). Pt/N-co doped TiO_2_ nanotubes and its photocatalytic activity under visible light. Appl. Surf. Sci..

[20] Zhao L., Xie Y., Lin Q., Zheng R., Diao Y. (2019). Preparation of C, N and P co-doped TiO_2_ and its photocatalytic activity under visible light. Funct. Mater. Lett..

[21] Lee J.Y., Park J., Cho J.H. (2005). Electronic properties of N- and C-doped TiO_2_. Appl. Phys. Lett..

[22] Kuo C., Chen W., Chen T. (2014). The electronic structure changes and the origin of the enhanced optical properties in N-doped anatase TiO_2_-A theoretical revisit. Int. J. Appl. Phys..

[23] Chen H., Li X., Wan R., Kao-Walter S., Lei Y., Leng C. (2018). A DFT study on modification mechanism of (N,S) interstitial co-doped rutile TiO_2_. Chem. Phys. Lett..

[24] Guo Y., Guo T., Chen J., Wei J., Bai L., Ye X., Ding Z., Xu W., Zhou Z. (2018). Synthesis of C-N-S co-doped TiO_2_ mischcrystal with an isobandgap characteristic and its photocatalytic activity under visible light. Catal. Sci. Technol..

[25] Mamakhel A., Yu J., Søndergaard-Pedersen F., Hald P., Iversen B.B. (2020). Facile synthesis of brookite TiO_2_ nano particles Electronic supplementary. Chem. Comm..

[26] Li W., Chu L., Gong X., Lu G. (2011). A comparative DFT study of adsorption and catalytic performance of Au nanoparticles at anatase and brookite TiO_2_ surfaces. Surf. Sci..

[27] Payne M.C., Teter M.P., Allan D.C., Arias T.A., Joannopoulos J.D. (1992). Iterative minimization techniques for ab initio total-energy calculations: Molecular dynamics and conjugate gradients. Rev. Mod. Phys..

[28] Wang Y., Zhang R., Li J., Li L., Lin S. (2014). First-principles study on transition metal-doped anatase TiO_2_. Nanoscale Res. Lett..

